# Effect of frailty on treatment, hospitalisation and death in patients with chronic heart failure

**DOI:** 10.1007/s00392-020-01792-w

**Published:** 2021-01-05

**Authors:** S. Sze, P. Pellicori, J. Zhang, J. Weston, I. B. Squire, A. L. Clark

**Affiliations:** 1 NIHR Leicester Biomedical Research Centre, University of Leicester, Glenfield Hospital, Groby Road, Leicester, LE3 9QP UK; 2 Department of Cardiology, Castle Hill Hospital, Hull York Medical School (At University of Hull), Kingston upon Hull, HU16 5JQ UK; 3 Robertson Centre for Biostatistics and Clinical Trials, University of Glasgow, Glasgow, G12 8QQ UK; 4 Faculty of Medical Science, Anglia Ruskin University, Cambridge, CB1 1PT UK

**Keywords:** Heart failure, Frailty, Cause of death, Cause of hospitalisation

## Abstract

**Background:**

Frailty is common in patients with chronic heart failure (CHF) and is associated with poor outcomes. The natural history of frail patients with CHF is unknown.

**Methods:**

Frailty was assessed using the clinical frailty scale (CFS) in 467 consecutive patients with CHF (67% male, median age 76 years, median NT-proBNP 1156 ng/L) attending a routine follow-up visit. Those with CFS > 4 were classified as frail. We investigated the relation between frailty and treatments, hospitalisation and death in patients with CHF.

**Results:**

206 patients (44%) were frail. Of 291 patients with HF with reduced ejection fraction (HeFREF), those who were frail (*N* = 117; 40%) were less likely to receive optimal treatment, with many not receiving a renin–angiotensin–aldosterone system inhibitor (frail: 25% vs. non-frail: 4%), a beta-blocker (16% vs. 8%) or a mineralocorticoid receptor antagonist (50% vs 41%). By 1 year, there were 56 deaths and 322 hospitalisations, of which 25 (45%) and 198 (61%), respectively, were due to non-cardiovascular (non-CV) causes. Most deaths (*N* = 46, 82%) and hospitalisations (*N* = 215, 67%) occurred in frail patients. Amongst frail patients, 43% of deaths and 64% of hospitalisations were for non-CV causes; 58% of cardiovascular (CV) deaths were due to advancing HF. Among non-frail patients, 50% of deaths and 57% of hospitalisations were for non-CV causes; all CV deaths were due to advancing HF.

**Conclusion:**

Frailty in patients with HeFREF is associated with sub-optimal medical treatment. Frail patients are more likely to die or be admitted to hospital, but whether frail or not, many events are non-CV.

**Graphical abstract:**

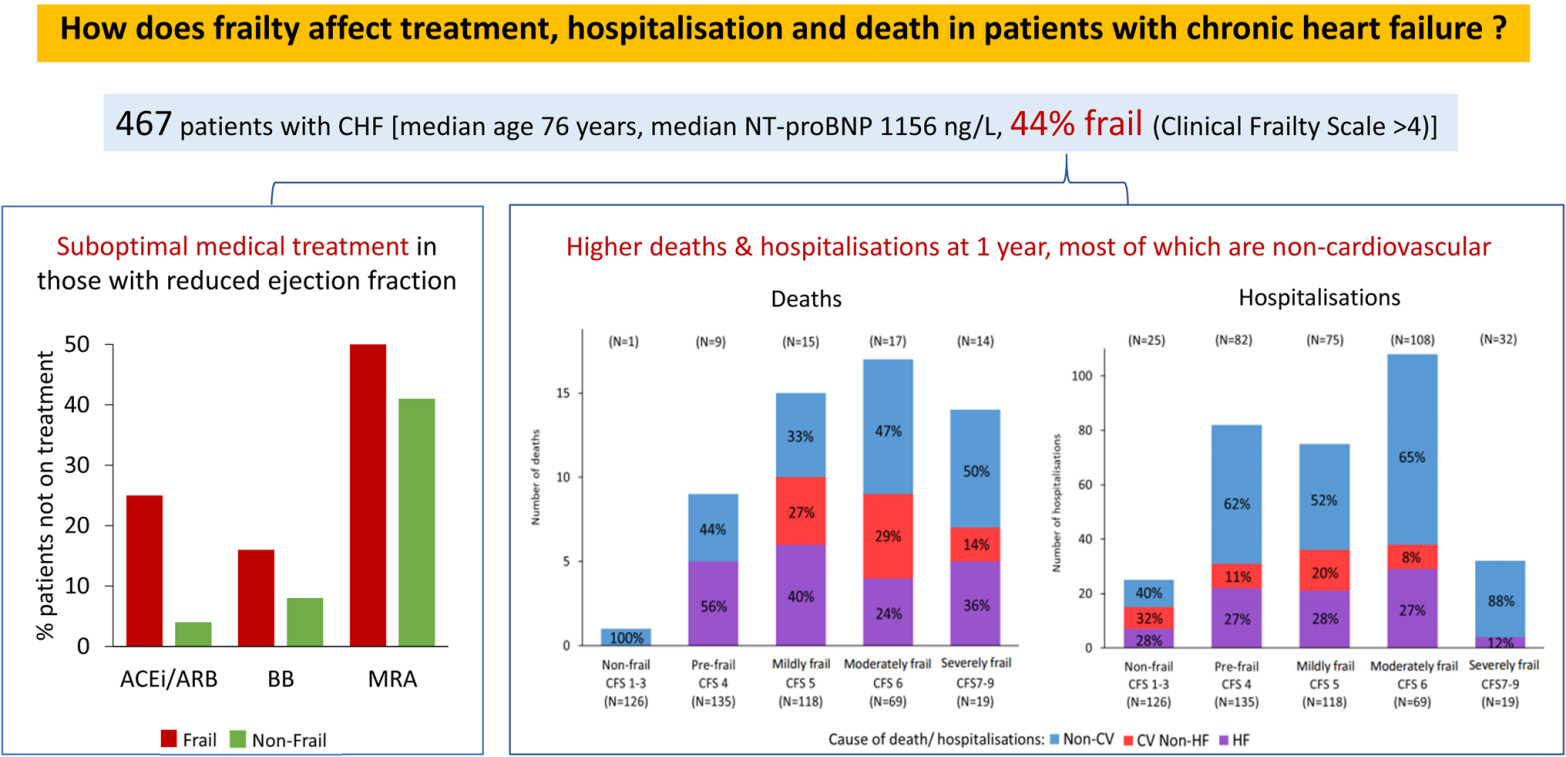

**Supplementary Information:**

The online version contains supplementary material available at 10.1007/s00392-020-01792-w.

## Introduction

Up to 50% of patients with chronic heart failure (CHF) are frail, and frailty is associated with an increased risk of morbidity and mortality [1, 2]. Frailty is a “multidimensional dynamic state, independent of age that makes the individual with CHF more vulnerable to the effect of stressors” [2]. The relation between CHF and frailty is complex: patients with CHF are up to six times more likely to be frail than those without CHF and frail individuals have a higher risk of developing CHF [3].

Clinical trials suggest that the vast majority of deaths in patients with CHF are cardiovascular, either due to progression of the underlying disease, or sudden, secondary to a fatal myocardial infarction or arrhythmias [4]. However, patients enrolled in clinical trials are poorly representative of those seen in routine clinical practice, who are usually older, with more comorbidities, and more likely to be frail [5]. The natural history of frail patients with CHF is unknown. A better understanding of their current management and of the reasons for their hospitalisations or death might help tailor future therapeutic strategies and enable more appropriate use of healthcare resources.

We therefore evaluated the causes of death and hospitalisations in ambulatory patients with CHF and their relation to frailty. We also assessed the association between frailty and rates of non-prescription of evidence-based therapies amongst patients with heart failure with reduced ejection fraction.

## Methods

### Study population

Between September 2016 and March 2017, we enrolled consecutive ambulatory patients with CHF who attended a community CHF clinic for a routine follow-up appointment. All patients had a pre-existing (> 1 year) clinical diagnosis of CHF, confirmed by either evidence of left ventricular systolic dysfunction on echocardiography (left ventricular ejection fraction (LVEF) < 40% or at least moderate left ventricular systolic dysfunction by visual inspection if LVEF was not calculated), defined as heart failure with reduced ejection fraction, HeFREF; or normal left ventricular systolic function (LVEF >/= 40%) and N-terminal pro-B-type natriuretic peptide (NTproBNP) > 400 ng/L, defined as heart failure with normal ejection fraction, HeFNEF [6].

During the visit, all patients had a full medical history and medication review, physical examination, blood tests (full blood count, urea and electrolytes and NT-proBNP), an electrocardiogram and a consultation with a heart failure specialist.

### Frailty assessment

During the same clinical visit, all patients were assessed for frailty using the Clinical Frailty Scale (CFS) by the same researcher (SS) (Online resource 1). CFS measures between 1 (very fit) and 9 (terminally ill). Subjects are scored according to their functional capacity, level of dependence and co-morbidities. For example, a patient with uncontrolled symptoms who is not frankly dependent is classified as vulnerable and scores 4 on the CFS; while an individual with limited dependence on others for instrumental activities of daily living including finances, transportation, heavy housework and medications will be classified as mildly frail and scores 5 on the CFS. Subjects with a CFS > 4 are classified as frail (Online resource 1) [7]. We stratified patients into five categories: non-frail (CFS 1–3), pre-frail (CFS 4), mildly frail (CFS 5), moderately frail (CFS 6) and severely frail (CFS ≥ 7). We chose CFS to evaluate frailty as it is a simple tool and has similar classification performance and prognostic value as alternative assessment tools taking much longer to perform [7, 8].

### Co-morbidities

Co-morbidities were measured using the Charlson co-morbidity index/score [9]. Hypertension was defined as systolic blood pressure ≥ 140 mmHg, diastolic blood pressure ≥ 90 mmHg or a previous clinical diagnosis [10]. Current haemoglobin (Hb) levels were used to define anaemia (Hb < 13.0 g/dL in men and < 12.0 g/dL in women) [11]. Diabetes mellitus was defined according to the guideline from Diabetes UK [12]. Patients consented to the use of electronic medical records to identify their previous medical history.

### Follow-up

All patients were followed up for a minimum of 1 year. Patients were followed up until 1st of August 2018. Our hospital is the only one in the region offering acute medical services. For the purpose of this study, we studied the *primary* cause of hospitalisations and death. Hospitalisations refer to non-elective admissions to hospital which require overnight stay.

Hospitalisation was ascertained using the hospital coding system, electronic medical records and discharge letters. Cardiovascular (CV) hospitalisations included hospitalisations secondary to decompensated CHF, acute coronary syndrome (ACS), arrhythmias, cerebrovascular accidents (CVA) and peripheral vascular disease (PVD). Other hospitalisations were regarded as non-cardiovascular (non-CV), including those related to acute kidney injury (AKI), falls, or infections.

Death adjudication followed a standard procedure. For those who died in hospital, the hospital notes were reviewed and cause of death was adjudicated. For those who died outside hospital, the patient’s general practitioner was contacted to obtain the cause of death recorded on death certificates. If this was unsuccessful, the cause of death was adjudicated based on previous medical records, recent hospitalisations and medical encounters. A detailed description of the adjudication process can be found in Online resource 2.

CV deaths included presumed sudden cardiac deaths or those caused by myocardial infarction (MI), terminal HF or CVA. Other deaths were regarded as non-CV, including those due to infection, malignancy or other comorbidities.

### Statistical analysis

Continuous data are expressed as a median (25th–75th centiles) and categorical data are expressed as %. Independent *t* tests and Mann–Whitney *U* tests were used to compare two continuous variables for normally and non-normally distributed data, respectively. The Chi-squared test was used to compare proportions between groups.

We studied the prescribing pattern of major classes of CHF medications (according to the European Society of Cardiology Guidelines) [8] in patients with HeFREF stratified by degree of frailty and correlated that with outcome. A detailed description of optimal doses of medications for patients with HeFREF is shown in online resource 3. Univariable and multivariable analyses with Cox proportional hazard regression were performed to determine the prognostic value of frailty. Bar charts are used to illustrate the proportion of HF vs. CV non-HF vs. non-CV deaths and hospitalisations in frail vs. non-frail patients. We also studied the detailed causes of CV and non-CV deaths and hospitalisations. We compared the causes of death and hospitalisations in patients stratified according to HF phenotype (HeFREF vs. HeFNEF), sex (male vs. female) and degree of frailty by CFS (non-frail, pre-frail, mildly frail, moderately frail and severely frail).

All statistical analyses were performed using SPSS 26 (SPSS INc.,Chicago, IL, USA) and the Stata (14th Version, StataCorp, TX, USA) statistical computer package. A two-tailed *P* value of < 0.05 was considered significant in all analyses.

The study conformed to the principles outlined in the Declaration of Helsinki and was approved by relevant ethical bodies. All subjects gave their written informed consent for their data to be used for research.

## Results

### Baseline characteristics

A total of 467 consecutive ambulatory patients with CHF was studied. Table 1 shows the baseline characteristics of the study population. The majority of patients were male and elderly; most patients had HeFREF (62%) with median NT-proBNP of 1156 (496–2463) ng/L; around 20% had severe symptoms [New York Heart Association (NYHA) class III/IV]. Almost half (44%) of the patients were frail.

**Table 1:** Baseline characteristics of patients with CHF by frailty status according to the CFS

	Entire cohort (*N* = 467)	CFS		*P* (Frail vs. non-frail)
		Non-frail (CFS ≤ 4) (*N* = 261)	Frail (CFS > 4) (*N* = 206)	
Demographics				
Age, years	76 (69–82)	72 (65–79)	80 (74–85)	< 0.001
Sex (male), %	67	72	60	0.005
HR (bpm)	70 (60–80)	70 (61–80)	70 (60–81)	0.70
BP systolic (mmHg)	139 (126–162)	140 (126–158)	137 (126–167)	0.55
BP diastolic (mmHg)	75 (66–83)	75 (67–83)	74 (65–83)	0.20
NYHA III/IV, %	22	10	38	< 0.001
HeFREF, %	62	67	57	0.03
HeFNEF, %	38	33	43	
Height (m)	1.68 (1.61–1.75)	1.70 (1.64–1.76)	1.66 (1.59–1.73)	< 0.001
Weight (kg)	83 (69–99)	86 (74–102)	77 (65–94)	< 0.001
BMI (kg/m^2^)	29.0 (25.0–33.2)	29.3 (26.0–34.2)	28.4 (24.2–32.4)	0.01
Comorbidities				
Charlson score	8 (6–10)	7 (5–9)	9 (8–11)	< 0.001
MI, %	42	43	42	0.80
PVD, %	15	12	19	0.03
HTN, %	67	64	70	0.17
CVA/TIA, %	15	10	22	< 0.001
AF, %	46	41	52	0.01
Diabetes, %	35	31	39	0.08
Dementia, %	10	2	21	< 0.001
COPD, %	30	24	37	0.002
Depression, %	20	15	27	0.001
Anaemia, %	47	36	60	< 0.001
Recurrent falls, %	37	18	61	< 0.001
Medications				
BB, %	84	88	79	0.01
ACEi/ARB, %	83	90	74	< 0.001
MRA, %	46	49	41	0.08
Digoxin, %	21	22	21	0.80
Loop diuretic, %	74	69	82	0.001
Statins, %	62	65	59	0.18
Pacemakers/devices, %	18	12	26	< 0.001
Blood tests				
NTproBNP (ng/L)	1156 (496–2463)	877 (372–1717)	1622 (784–3296)	< 0.001
Hb (g/L)	131 (118–142)	135 (124–145)	123 (114–136)	< 0.001
Na (mmol/L)	137 (135–138)	137 (135–139)	136 (134–138)	0.04
K (mmol/L)	4.4 (4.2–4.7)	4.5 (4.2–4.7)	4.4 (4.1–4.7)	0.09
eGFR (mL/min per 1.73 m^2^)	55 (40–73)	60 (45–76)	50 (33–67)	< 0.001
Creatinine (µmol/L)	103 (84–131)	99 (83–123)	110 (88–141)	0.001
1 year outcomes				
All-cause deaths, %	12	4	22	< 0.001
≥ 1 hospitalisations, %	32	21	47	< 0.001

*HF* heart failure, *HR* heart rate, *BP* blood pressure, *NYHA* new York heart association, *HeFREF* heart failure with reduced ejection fraction, *HeFNEF* heart failure with normal ejection fraction, *BMI* body mass index, MI = myocardial infarction, *PVD* peripheral vascular disease, *HTN* hypertension, *CVA/TIA* cerebrovascular accident/ transient ischaemic attack, *COPD* chronic obstructive pulmonary disease, *BB* beta-blocker, *ACEi* angiotensin converting enzyme inhibitor, *ARB* angiotensin receptor blocker, *MRA* mineralocorticoid receptor antagonist, NTproBNP = N-terminal pro-B-type natriuretic peptide, Hb = haemoglobin, Na = sodium, K = potassium, eGFR = estimated glomerular filtration rate

Compared to patients who were not frail, those who were frail were older, more likely to be female and more likely to have HeFNEF. They had more comorbidities and worse renal function with lower body mass index (BMI) and haemoglobin. They also had more severe symptoms and higher NT-proBNP (Table 1).

### Medication in patients with HeFREF and degree of frailty

**Fig. 1:**
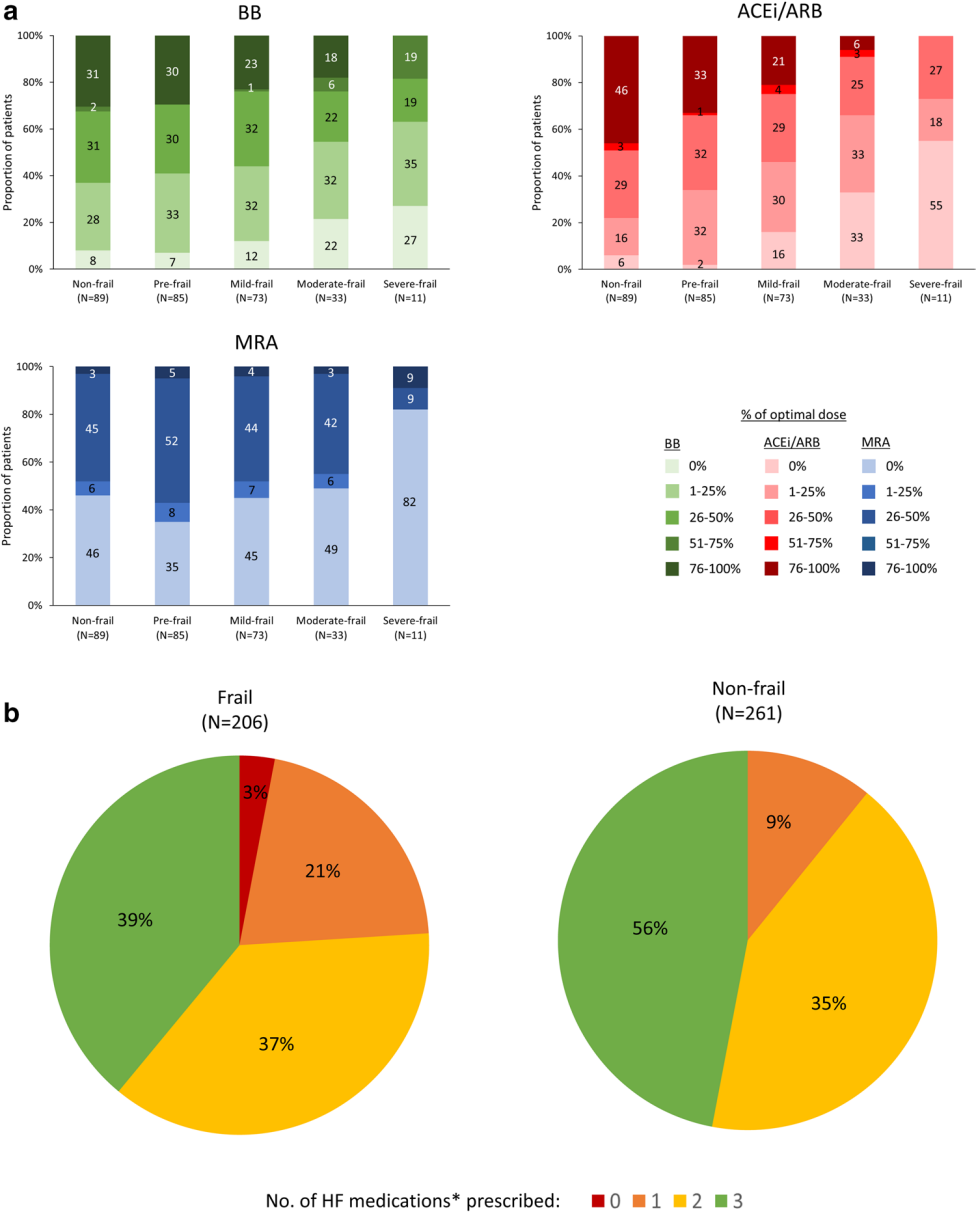
**a**: Major classes of CHF medications prescribed in patients with HeFREF according to frailty categories (upper left: Beta-blockers (*BB*); upper right: ACEi/ARB; lower left: MRA). The numbers within bars represent the percentage of patients on different dose ranges of CHF medications in each frailty category. **b**: Number of HF medications prescribed for patients with HeFREF according to frailty status. *HF medications refer to ACEi/ARB, beta-blockers and MRA

Figure 1a shows the three major classes of CHF medications prescribed in patients with HeFREF stratified according to the degree of frailty. Compared to non-frail patients, frail patients were less likely to be prescribed ACEi/ARB, beta-blockers and MRA (Fig. 1a-b). Those who did receive treatment were more likely to receive sub-optimal doses (Fig. 1a). As frailty worsened, the likelihood of receiving sub-optimal doses increased.

### Frailty is an independent predictor of poor outcome

Worsening frailty, as determined by increasing CFS score, was associated with increased risk of all-cause mortality and combined all-cause mortality/all-cause hospitalisation at 1 year in both univariable and multivariable Cox regression analyses after adjustment of age, BMI, NYHA class, Charlson score, log[NTproBNP], haemoglobin and estimated glomerular filtration rate (Online resource 4a and 4b).

### Cause of death

**Fig. 2:**
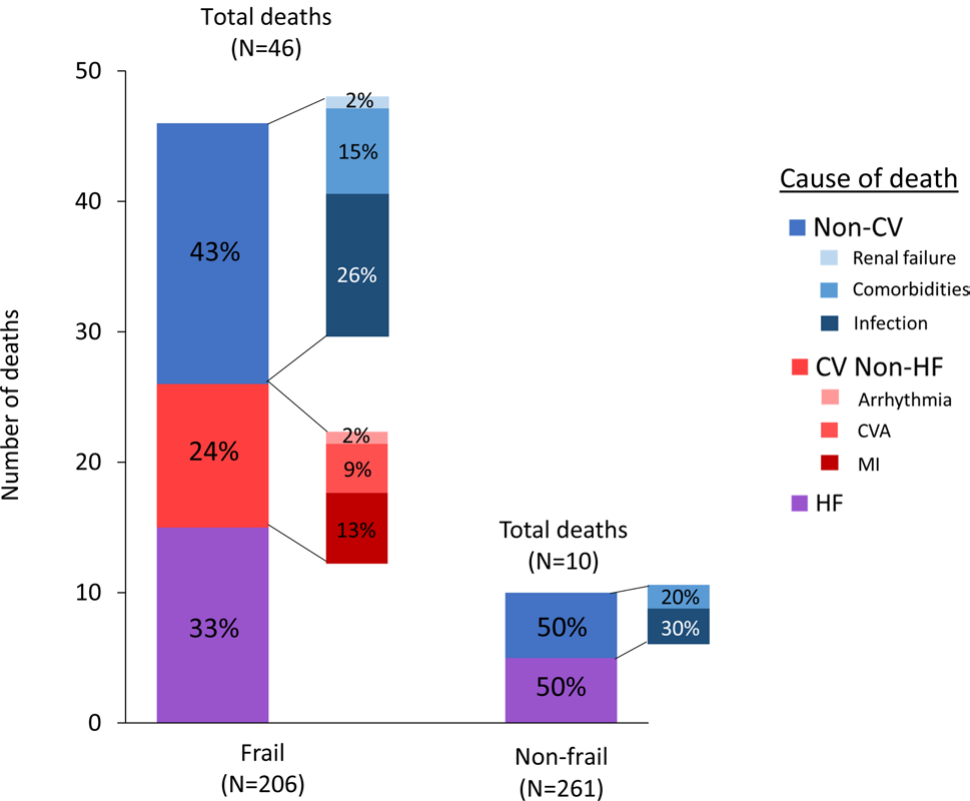
Cause of death at 1 year in frail vs. non-frail patients

**Fig. 3:**
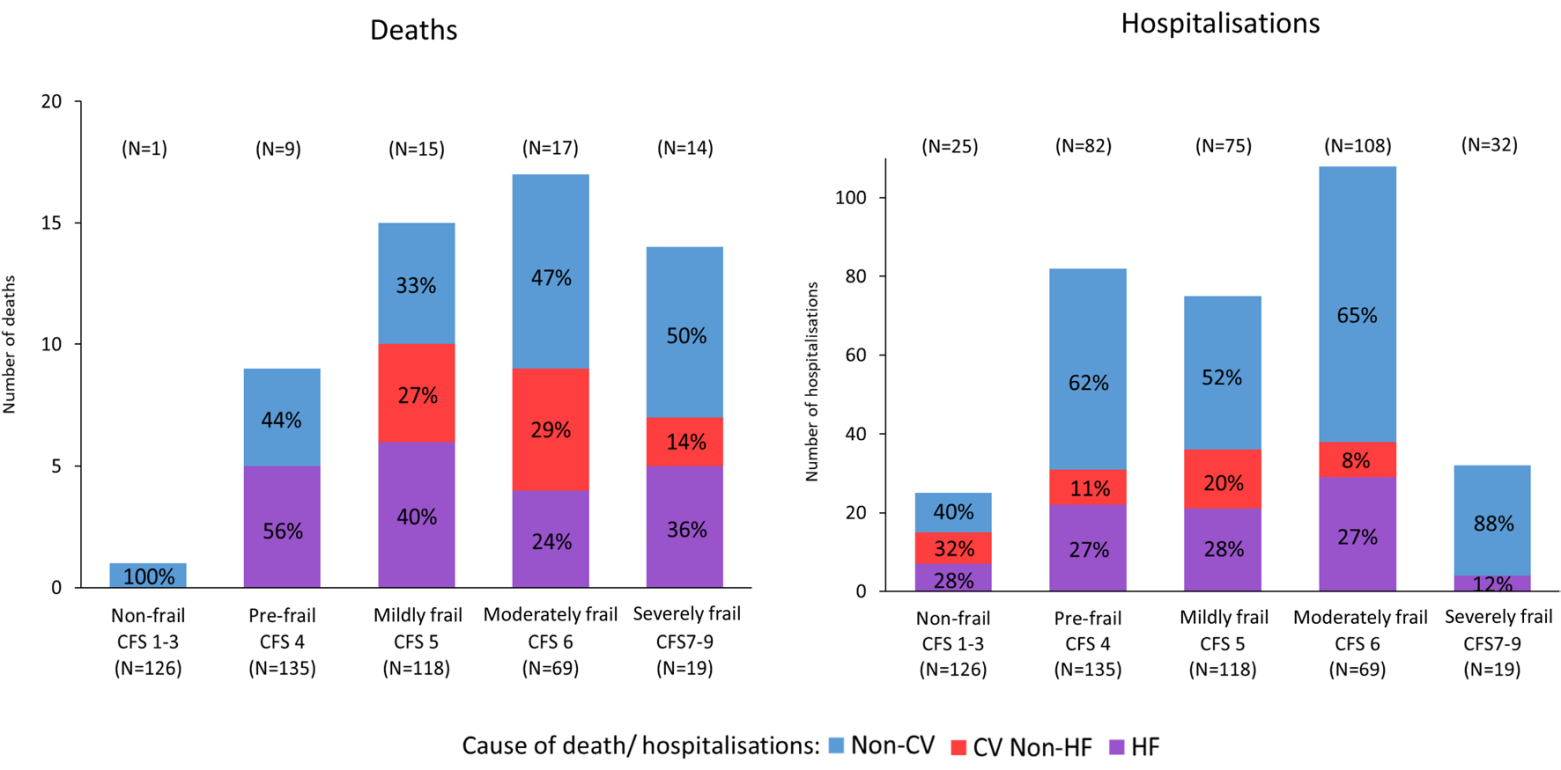
HF vs. CV non-HF vs non-CV deaths (left panel) and hospitalisations (right panel) at 1 year in frail vs. non-frail patients according to degree of frailty

By 1-year follow-up, 56 (12%) patients had died, representing 22% (*N* = 46) of the frail patients and 4% (*N* = 10) of the non-frail (Fig. 2). In frail patients who died, the cause was non-CV in 43%, compared with 50% amongst non-frail patients (*P* = 0.71). Regardless of frailty status, infection was the commonest cause of non-CV deaths, while advancing HF was the commonest cause of CV death. The primary cause of death was similar between patients with different HF phenotypes and sexes (Online resource 5a). The proportion of non-CV deaths increased with increasing severity of frailty (Fig. 3).

### Cause of hospitalisation

**Fig. 4:**
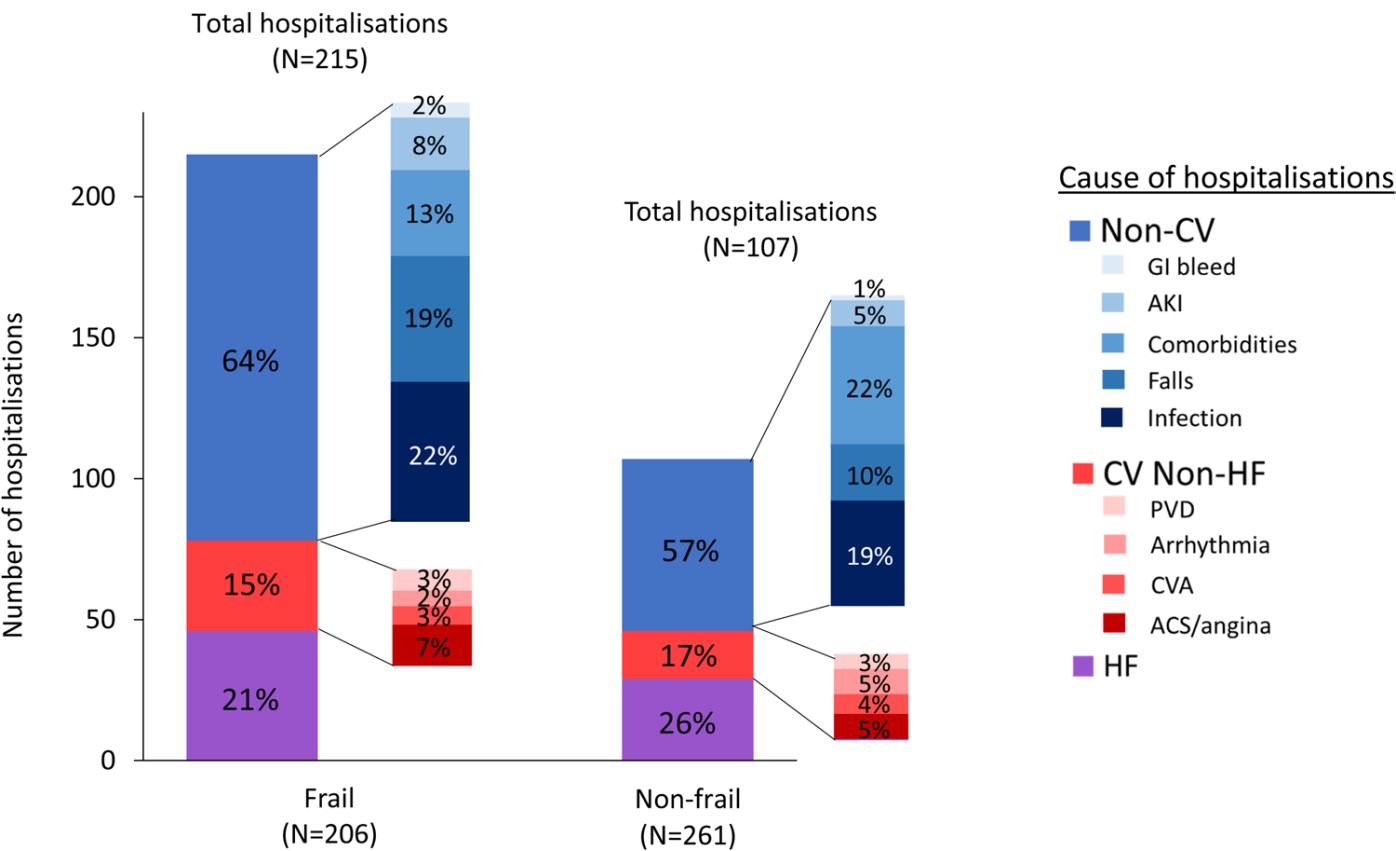
Cause of hospitalisations at 1 year in frail vs. non-frail patients

By 1-year follow-up, there were 322 non-elective hospitalisations; 215 events occurred in 96 (47%) frail patients and 107 events in 54 (21%) non-frail patients (Fig. 4). In frail patients, 64% of hospitalisations were due to non-CV causes compared with 57% in non-frail patients (*P* = 0.25). Regardless of frailty status, decompensated HF was the commonest cause of CV hospitalisations. Of non-CV hospitalisations, falls were more common in frail patients, particularly in those with HeFNEF and in female patients, while admissions related to comorbidities were more common in non-frail patients (Fig. 4 and Online resource 5b). The proportion of non-CV hospitalisations increased with increasing severity of frailty (Fig. 3).

### Recurrent hospitalisations

**Fig. 5:**
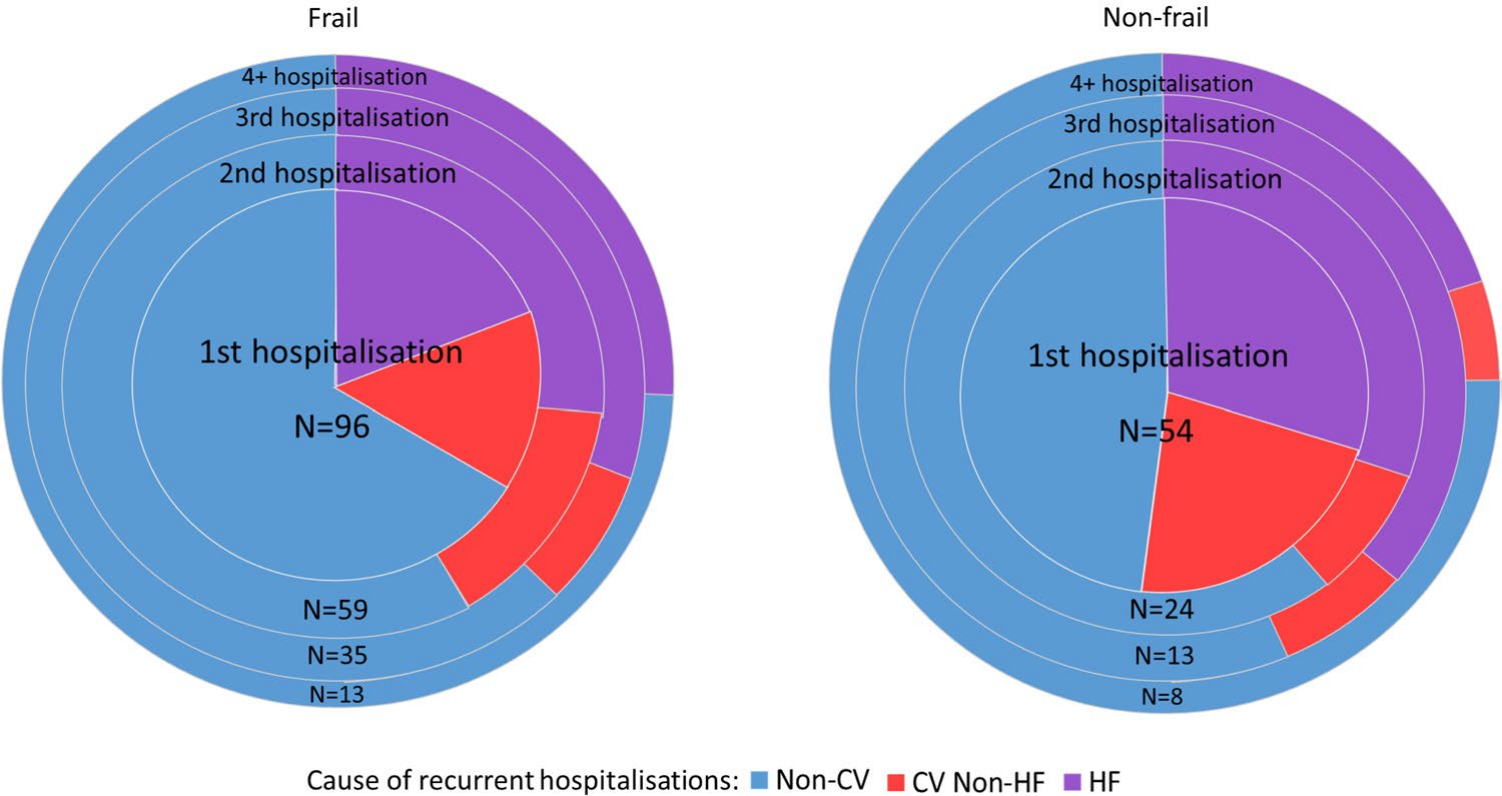
Cause of recurrent hospitalisations at 1 year in frail vs. non-frail patients with CHF

The average numbers of hospitalisations per patient per 365 days of follow-up in frail and non-frail patients are 1.0 and 0.4, respectively (*P* < 0.001). Of patients who were frail (*N* = 206), 96 experienced hospitalisation, of whom many had recurrent hospitalisations: 2nd hospitalisations *N* = 59; 3rd hospitalisations *N* = 35 and ≥ 4 hospitalisations *N* = 13 (Fig. 5). The majority of recurrent hospitalisations were due to non-CV causes (Fig. 5). The causes of recurrent hospitalisations in HeFREF and HeFNEF patients are shown in Online resource 6.

## Discussion

Our study is the first to examine the relation between frailty and causes of death, first and recurrent hospitalisations in a well-characterised cohort of ambulatory patients with CHF. We found that during the first year of follow-up, compared to non-frail individuals, frail patients experienced a far higher rate of death and hospitalisations, a large proportion of which were for non-CV causes. The proportion of events due to non-CV reasons was high and similar in frail and non-frail patients when frailty was treated as a categorical variable, but as the frailty status of a patient worsened, the proportion of non-CV deaths and hospitalisations increased. We also found that frail patients with HeFREF were less likely to receive (or receive optimal doses of) guideline-recommended CHF therapy.

Before the era of ACE inhibitors, it was estimated that 90% of the total deaths in patients with CHF were from CV causes [14, 15]. In contrast, we have found that approximately half of all deaths were due to non-CV causes. Our findings are similar to those from a Spanish cohort study which explored the cause of death in 1876 ambulatory patients with CHF (75% male, median age 66 years, LVEF < 50%) between 2002 and 2018 [16]. During a median follow-up of 4.2 years, there were 935 deaths, of which 40% were non-CV and 60% were CV. The authors also noticed a progressive increase in the rate of non-CV death over time, representing more than 50% of total deaths from 2015 onwards.

There has been a remarkable shift in the causes of death in CHF populations over the past two decades. In particular, there has been a significant reduction in sudden death and a concomitant increase in non-CV deaths [17]. There are several possible explanations. First, as a result of improvement in the management of conditions leading to CHF, such as coronary artery disease and hypertension, and major advances in treatment for HeFREF, an increasing proportion of patients with CHF is frail and elderly. Almost half (44%) of the patients we studied were frail. Multi-morbidity is extremely common in patients with CHF. In the UK, 79% of patients with CHF have three or more comorbidities [18]. Non-CV diseases, such as malignancies, chronic kidney disease, cognitive decline and depression, are very common amongst patients with CHF [18]. Second, as the population ages, the proportion of patients with CHF who have HeFNEF increases, these patients have a particularly high burden of comorbidities and are thus at high risk of non-CV deaths and hospitalisations.

CHF is a cardiogeriatric syndrome, but frail elderly patients are under-represented in the vast majority of clinical trials in CHF [19]. Part of the reason are the efforts of investigators to make sure that any treatment effects are not swamped by the effects of comorbidities; and partly because of the inability (perceived or otherwise) of older, frailer patients to cope with frequent study-related visits and procedures. To take as an example, the recent PARADIGM-HF trial [20]: applying the inclusion and exclusion criteria to the population we report here, 83% of frail patients would have been excluded compared to 65% of non-frail patients (*p* < 0.001) (Online resource 7). The consequence is that it can be difficult to ascertain what the effect of specific treatments might be in older, frailer patients. Very few studies have evaluated treatments specifically in elderly patients. Examples include the SENIORS trial, [21] which studied the effect of nebivolol on outcomes in patients with CHF > 70 years; and CIBIS-ELD trial, [22] which compared the tolerance of bisoprolol and carvedilol in patients with CHF > 65 years. In recent trials, such as DAPA-HF and PARADIGM-HF, only a minority of patients enrolled were > 75 years of age (24% and 19%, respectively) [20, 23]. However, there was no evidence of lesser benefit from sacubitril/valsartan or dapaglifozin in older patients according to these trials [20, 23].

The ESC/HFA guidelines recommend evidence-based pharmacotherapy for HeFREF patients irrespective of age. [13] However, we found that a large proportion of frail patients were prescribed sub-optimal CHF therapy. The worse the frailty status, the less likely it was for patients to receive optimal CHF therapy. Reasons include the possibility of more side effects or greater intolerance to medications in frailer patients; or physician inertia in initiating and up-titrating guideline-recommended medications in older patients. The presence of comorbidity, such as renal impairment, is increasingly common as the degree of frailty increases. Loop diuretics, on the other hand, are commonly used in frail patients, which might reflect attempts to treat severe symptoms and signs due to congestion [24].

The prevalence of frailty will increase as the population ages. The management of frail patients with CHF will become a significant medical challenge. We have shown that although frail patients are sub-optimally treated for HF, they are less likely to die of HF as frailty increases. Aggressive HF treatment is perhaps less and less important with increasing frailty; interventions to address frailty rather than to address HF are what is needed for these patients. Holistic care using a patient-centred approach is required. Common issues, such as malnutrition [25], cognitive decline, reduced mobility and depression [26], should be assessed and addressed as early as possible. More emphasis should be put on exploring what is important to the patient and palliative care should be considered according to the individual’s wishes [27].

### Study limitations

This study has some limitations. First, the study was conducted in a single centre in the UK with limited sample size, and thus external validation of our results from other populations with different healthcare and social systems is needed.

Second, we have only studied one of the most commonly used frailty tools. A large number of frailty screening and assessment tools have been proposed and identified patients at risk of adverse outcome in other clinical scenarios [28]. However, we have previously shown that CFS identifies frailty [1] and provides comparable prognostic information to assessment tools taking longer to perform [8].

We have clear in-house guidance to adjudicate events in a coherent and objective manner, but we recognise that accuracy of adjudication might have been sub-optimal. Patients with CHF are mostly elderly with multiple comorbidities, and some events, such as pulmonary embolism or infections, might have been missed as a cause of death [29]. Whilst autopsy is the gold standard for determining cause of death, they are rarely performed, and may not provide conclusive evidence [30]. In our department, event adjudication was performed by experienced physicians trained for the purpose. In cases where the cause of hospitalisation was uncertain, to ensure the accuracy of the adjudication process, the research medical team reviewed all medical entries, blood tests and radiological evidences available during that hospital admission to determine the primary cause of hospitalisation.

Although we collected data regarding medication use in our cohort, we did not study the reasons for under-prescriptions and missed/ failed up-titration of anti-HF medications. Further studies are needed to identify factors associated with under prescription of medication in patients with HeFREF, with a particular focus on the role of frailty in drug intolerance and non-adherence, so that targeted interventions can be developed to improve prognosis of this high-risk population.

## Conclusion

Frailty in patients with HeFREF is associated with sub-optimal medical treatment. Frail patients are more likely to die or be admitted to hospital, but whether frail or not, many events are non-CV, suggesting that non-HF interventions might be important. A holistic and patient-centred approach is needed to address the various healthcare needs of patients with CHF, especially in those living with frailty.

## Supplementary Information

Below is the link to the electronic supplementary material.

Supplementary file1 (DOCX 565 KB)

Supplementary file2 (DOCX 14 KB)

Supplementary file3 (DOCX 14 KB)

Supplementary file4 (DOCX 21 KB)

Supplementary file5 (PDF 139 KB)

Supplementary file6 (PDF 145 KB)

Supplementary file7 (PDF 88 KB)

Supplementary file8 (DOCX 26 KB)
