# Supplementary figures and images for: Effect of frailty on treatment, hospitalisation and death in patients with chronic heart failure
Source: Clin Res Cardiol. 2021 Jan 5;110(8):1249–58. doi: 10.1007/s00392-020-01792-w (PMC8318949; doi:10.1007/s00392-020-01792-w)

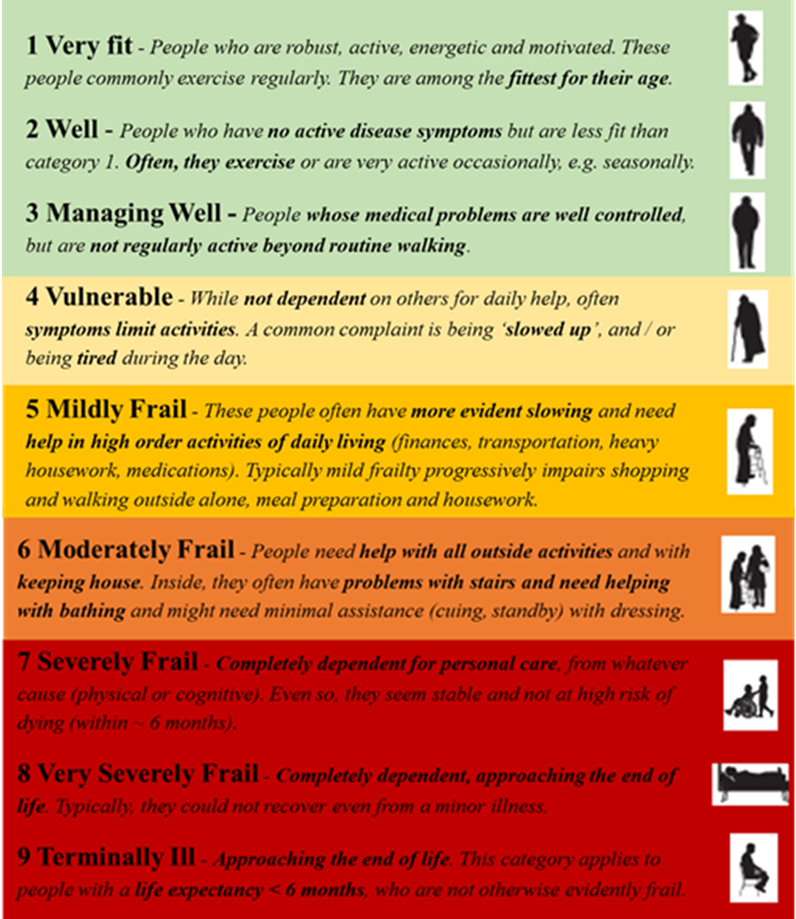
Online resource 1. Evaluation of frailty by Clinical Frailty Scale.

Supplement: Supplementary file 1 — Supplementary file1 (DOCX 565 KB) [file 392_2020_1792_MOESM1_ESM.docx]

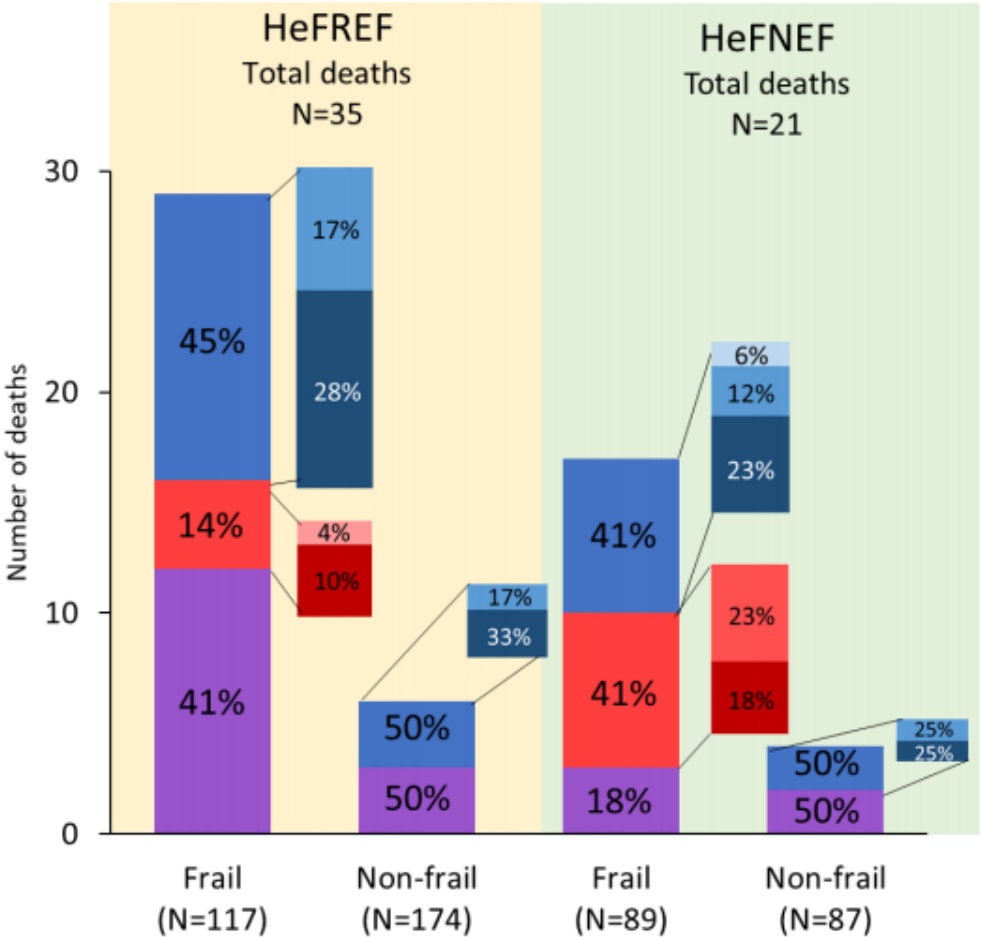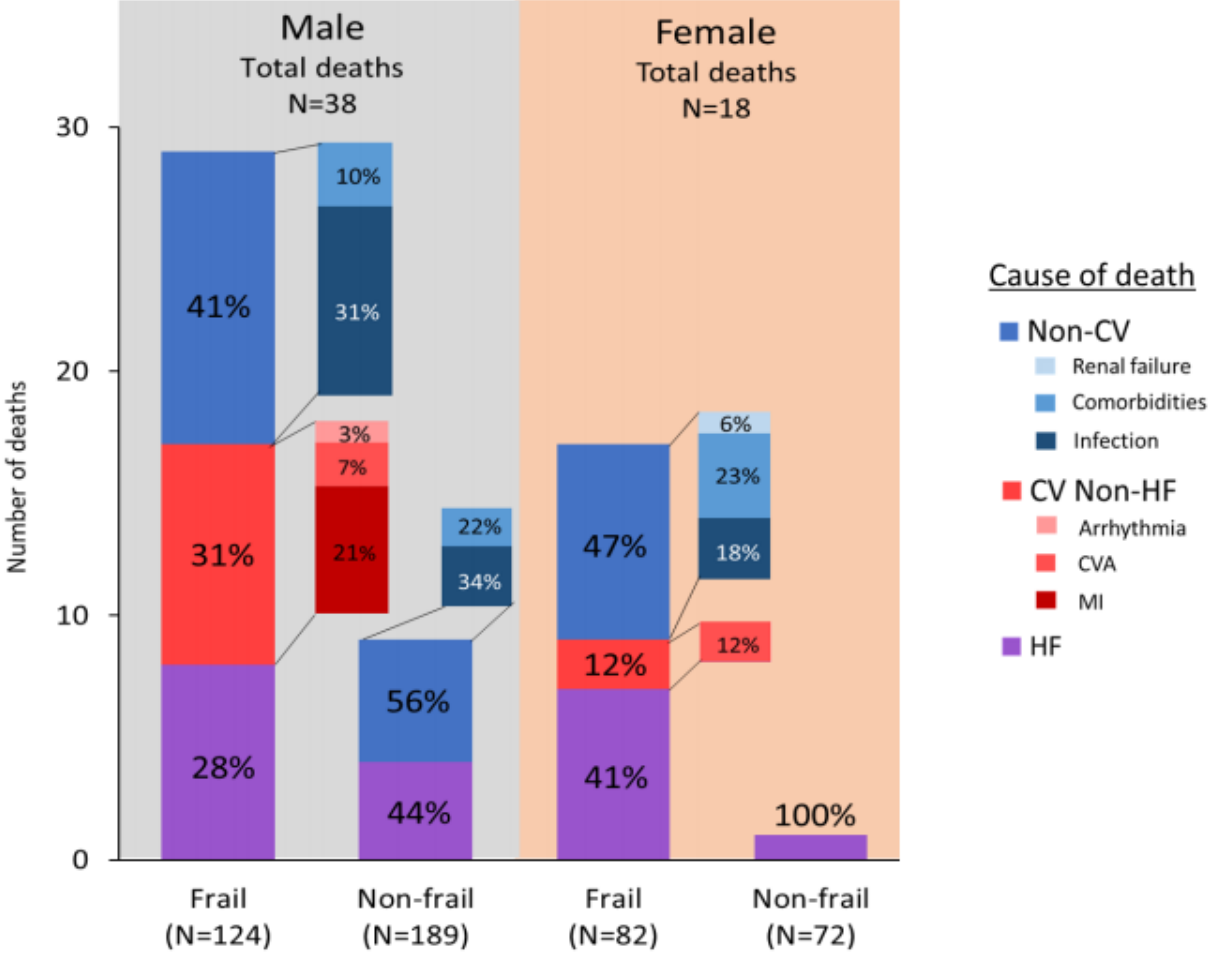

Supplement: Supplementary file 5 — Supplementary file5 (PDF 139 KB) [file 392_2020_1792_MOESM5_ESM.pdf]

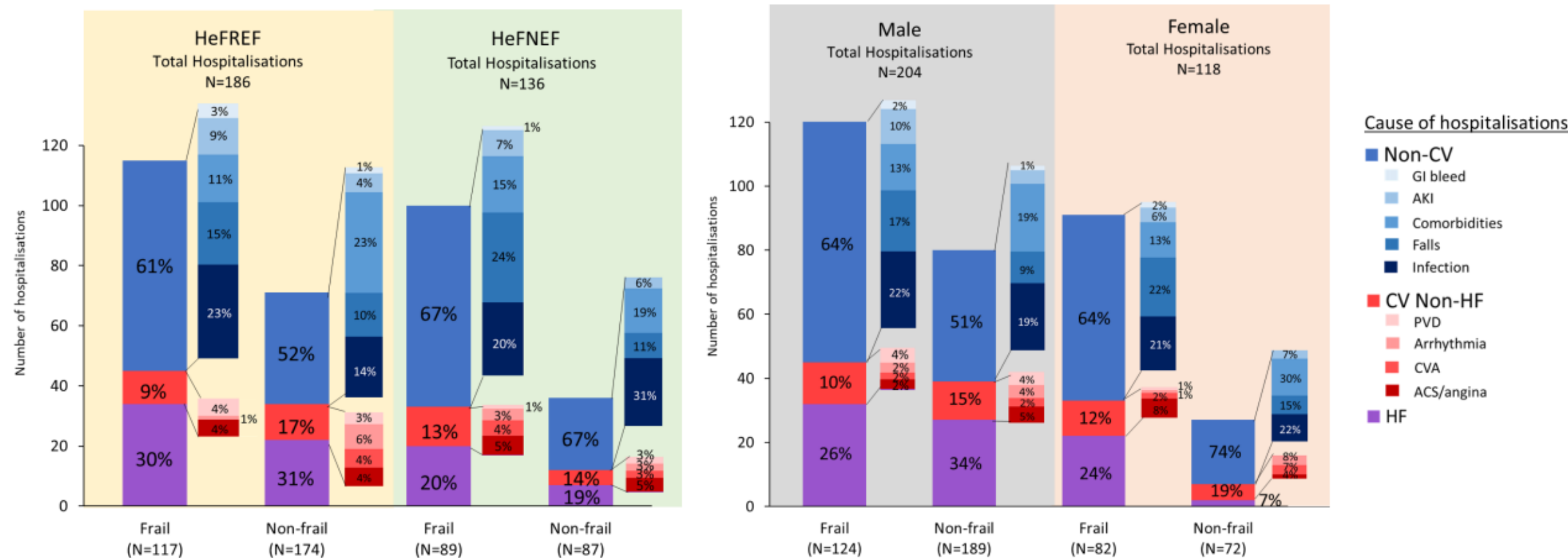

Supplement: Supplementary file 6 — Supplementary file6 (PDF 145 KB) [file 392_2020_1792_MOESM6_ESM.pdf]

HeFREF

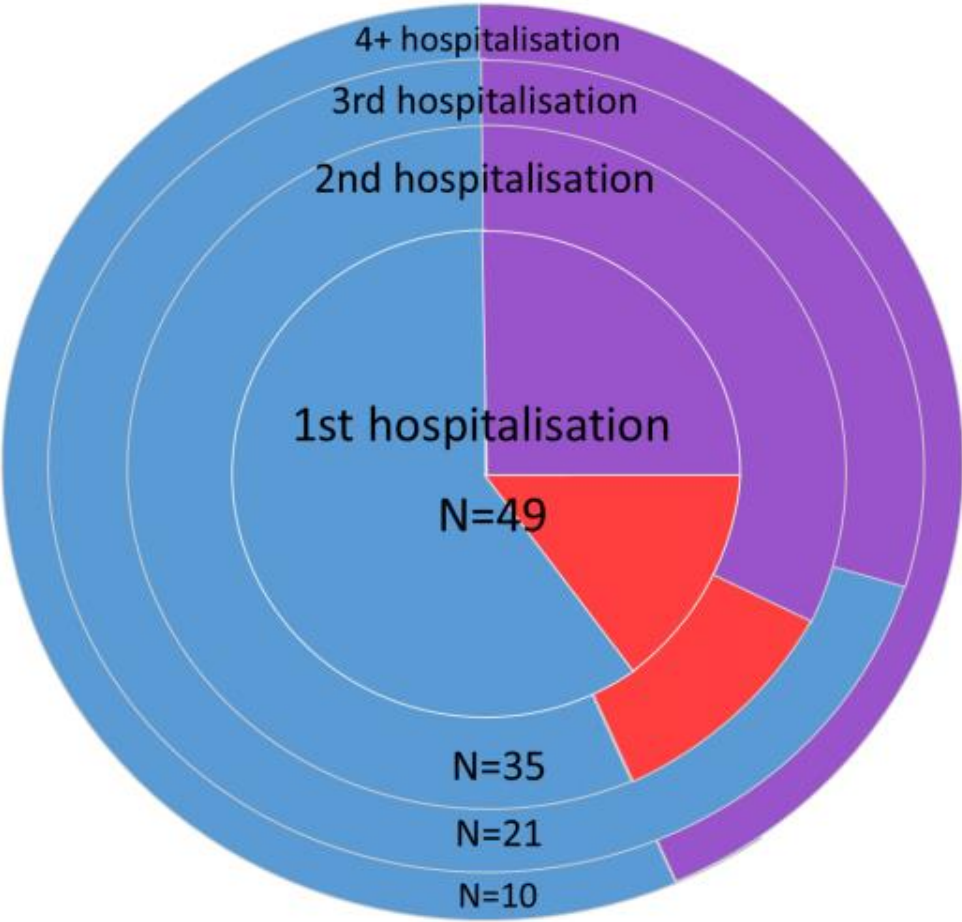

HeFNEF

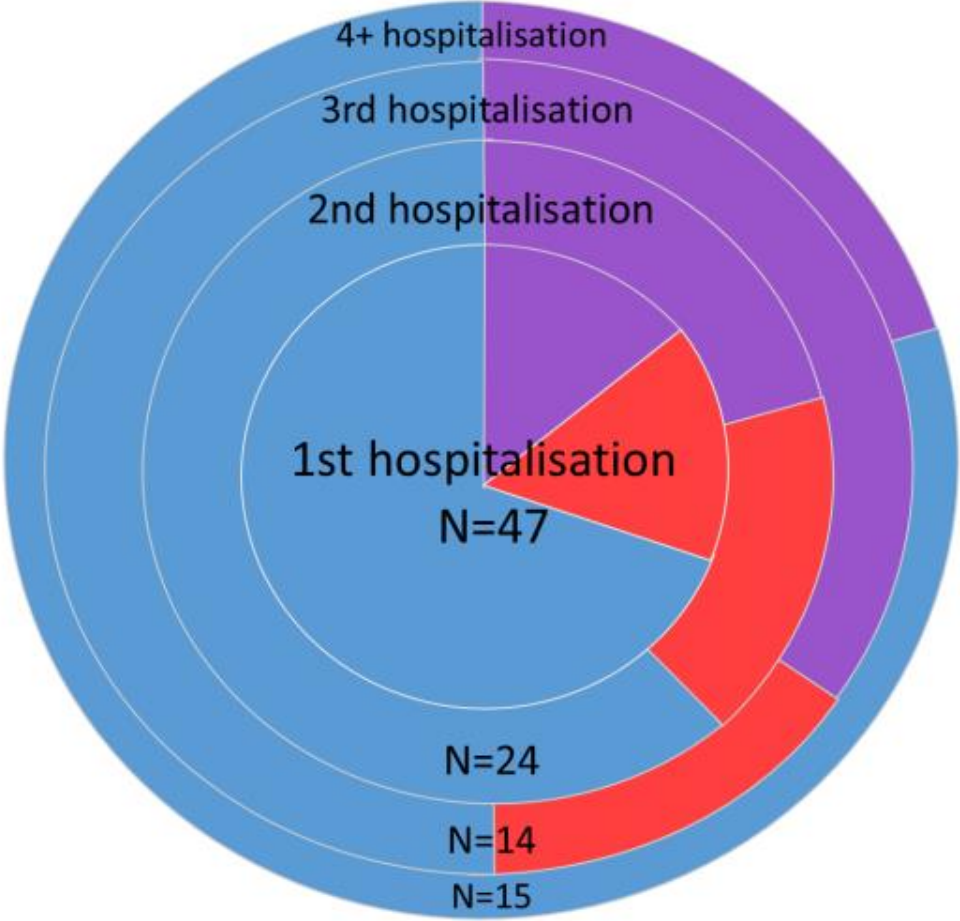

Supplement: Supplementary file 7 — Supplementary file7 (PDF 88 KB) [file 392_2020_1792_MOESM7_ESM.pdf]
