# Supplementary material for: Effect of frailty on treatment, hospitalisation and death in patients with chronic heart failure
Source: Clin Res Cardiol. 2021 Jan 5;110(8):1249–58. doi: 10.1007/s00392-020-01792-w (PMC8318949; doi:10.1007/s00392-020-01792-w)
Supplement: Supplementary file 2 — Supplementary file2 (DOCX 14 KB) [file 392_2020_1792_MOESM2_ESM.docx]

# Online resource 2: Adjudication process

# Cause of death

Briefly, the primary cause of death was ascertained using blood results, diagnostic investigations and correspondence on the primary and secondary care electronic records (updated systematically using an NHS electronic database), autopsy reports and death certificates.

For patients who died in hospital, the research medical team reviewed all medical entries, blood tests and radiological evidences available during the hospital admission, in conjunction with information reported by the last treating physician on the death certificate.

For patients who died out of hospital, the research medical team contacted the deceased’s general practitioners to obtain the cause of death recorded on death certificates. If this was unsuccessful, the cause of death was adjudicated by the medical team based on previous medical records, recent hospitalisations and medical encounters (including related clinic letters and prescription charts). For example, if a patient had recurrent hospital admissions with decompensated HF, or was persistently in New York Heart Association (NYHA) class III or IV in clinic in the months prior to death, then the primary cause of death was adjudicated to be terminal HF. Similarly, if the patient had been referred to palliative care or long-term care facility after a hospitalisation due to terminal illness such as cancer or HF, then that illness was adjudicated to be the cause of death.

Cardiovascular (CV) deaths included sudden arrhythmic deaths or those caused by myocardial infarction (MI), terminal HF or cerebrovascular accidents (CVA). Other deaths were regarded as non-cardiovascular (non-CV), including those due infection, malignancy or other end-stage comorbidities.

# Cause of hospitalisation

Hospitalisation was ascertained by using the hospital coding system, electronic medical records and discharge letters. The cause of hospitalisation reported in this study is the primary cause of hospitalisation. In cases where multiple causes of hospitalisation were recorded, the research medical team reviewed all medical entries, blood tests and radiological evidences available during that hospital admission to determine the primary cause of hospitalisation. CV hospitalisations included hospitalisations secondary to decompensated HF, acute coronary syndrome (ACS), arrhythmias, CVA and peripheral vascular disease (PVD). Other hospitalisations were regarded as non CV, including those related to acute kidney injury (AKI), falls, or infections.
