# Supplementary material for: Effect of frailty on treatment, hospitalisation and death in patients with chronic heart failure
Source: Clin Res Cardiol. 2021 Jan 5;110(8):1249–58. doi: 10.1007/s00392-020-01792-w (PMC8318949; doi:10.1007/s00392-020-01792-w)
Supplement: Supplementary file 3 — Supplementary file3 (DOCX 14 KB) [file 392_2020_1792_MOESM3_ESM.docx]

Online resource 3: Optimal doses of medications for HeFREF as per European Society of Cardiology guidelines.

|  | Optimal dose (per day) |
| --- | --- |
| Angiotensin converting enzyme inhibitors (ACEi) | |
| Ramipril | 10mg |
| Enalapril | 20mg |
| Lisinopril | 20mg |
| Perindopril | 4mg |
| Angiotensin receptor blockers (ARB) | |
| Candesartan | 32mg |
| Valsartan | 320mg |
| Losartan | 150mg |
| Beta-blockers | |
| Bisoprolol | 10mg |
| Carvedilol | 50mg |
| Metoprolol | 200mg |
| Nebivolol | 10mg |
| Mineralocorticoid receptor antagonist (MRA) | |
| Spironolactone | 50mg |
| Eplerenone | 50mg |
