# Supplementary material for: Effect of frailty on treatment, hospitalisation and death in patients with chronic heart failure
Source: Clin Res Cardiol. 2021 Jan 5;110(8):1249–58. doi: 10.1007/s00392-020-01792-w (PMC8318949; doi:10.1007/s00392-020-01792-w)
Supplement: Supplementary file 4 — Supplementary file4 (DOCX 21 KB) [file 392_2020_1792_MOESM4_ESM.docx]

Online resource 4a: Univariable and multivariable cox regression analyses for predicting all-cause mortality at 1 year.

| Worse outcome per unitary increase | **All-cause mortality** | | | | | |
| --- | --- | --- | --- | --- | --- | --- |
|  | **Univariable** | | | **Multivariable** | | |
|  | HR (95% CI) | Wald ꭓ^2^ | P | HR (95% CI) | Wald ꭓ^2^ | P |
| Age (years) | 1.08 (1.05-1.12) | 22.7 | <0.001 | 1.00 (0.96-1.04) | 0.01 | 0.99 |
| BMI (kg/m^2^) | 0.94 (0.89-0.98) | 7.6 | 0.006 | 0.97 (0.92-1.02) | 1.40 | 0.24 |
| Rhythm (AF vs SR) | 2.41 (1.39-4.19) | 9.7 | 0.002 | 1.43 (0.79-2.62) | 1.38 | 0.24 |
| NYHA (III/IV vs I/II) | 2.92 (1.72-4.96) | 15.7 | <0.001 | 0.88 (0.47-1.60) | 0.19 | 0.67 |
| Charlson Score | 1.39 (1.25-1.53) | 39.8 | <0.001 | 1.06 (0.91-1.24) | 0.52 | 0.47 |
| Log [NT-proBNP] | 8.21 (4.41-15.28) | 44.0 | <0.001 | 2.82 (1.36-5.85) | 7.73 | 0.005 |
| Hb (g/L) | 0.96 (0.95-0.98) | 22.5 | <0.001 | 1.00 (0.98-1.02) | 0.11 | 0.74 |
| eGFR (mL/min per 1.73 m^2^ ) | 0.97 (0.95-0.98) | 20.8 | <0.001 | 0.99 (0.97-1.01) | 1.75 | 0.19 |
| **CFS** | **2.72 (2.12-3.50)** | **61.8** | **<0.001** | **2.12 (1.54-2.92)** | **21.55** | **<0.001** |

Online resource 4b: Univariable and multivariable cox regression analyses for predicting combined all-cause mortality/ hospitalisation at 1 year.

| Worse outcome per unitary increase | **Combined all-cause mortality/ hospitalisation** | | | | | |
| --- | --- | --- | --- | --- | --- | --- |
|  | **Univariable** | | | **Multivariable** | | |
|  | HR (95% CI) | Wald ꭓ^2^ | P | HR (95% CI) | Wald ꭓ^2^ | P |
| Age (years) | 1.05 (1.04-1.07) | 37.1 | <0.001 | 1.00 (0.98-1.02) | 0.08 | 0.78 |
| BMI (kg/m^2^) | 0.97 (0.94-0.99) | 7.7 | 0.006 | 0.98 (0.95-1.01) | 2.6 | 0.11 |
| NYHA (III/IV vs I/II) | 3.03 (2.22-4.14) | 48.8 | <0.001 | 1.40 (0.98-1.99) | 3.4 | 0.06 |
| Charlson Score | 1.32 (1.24-1.39) | 85.7 | <0.001 | 1.14 (1.04-1.24) | 8.6 | 0.003 |
| Log [NT-proBNP] | 3.26 (2.33-4.57) | 47.3 | <0.001 | 1.48 (1.01-2.16) | 4.1 | 0.04 |
| Hb (g/L) | 0.96 (0.95-0.97) | 55.9 | <0.001 | 0.98 (0.97-0.99) | 9.9 | 0.002 |
| eGFR (mL/min per 1.73 m^2^ ) | 0.98 (0.97-0.98) | 38.4 | <0.001 | 1.00 (0.99-1.01) | 0.03 | 0.88 |
| **CFS** | **1.97 (1.72-2.25)** | **96.7** | **<0.001** | **1.39 (1.17-1.66)** | **14.1** | **<0.001** |

BMI= body mass index, AF= atrial fibrillation, SR= sinus rhythm, NYHA= New York Heart Association, NTproBNP= N-terminal pro-B-type natriuretic peptide, Hb= haemoglobin, eGFR = estimated glomerular filtration rate, CFS = clinical frailty scale
