# Supplementary material for: Effect of frailty on treatment, hospitalisation and death in patients with chronic heart failure
Source: Clin Res Cardiol. 2021 Jan 5;110(8):1249–58. doi: 10.1007/s00392-020-01792-w (PMC8318949; doi:10.1007/s00392-020-01792-w)
Supplement: Supplementary file 8 — Supplementary file8 (DOCX 26 KB) [file 392_2020_1792_MOESM8_ESM.docx]

Online resource 7: Inclusion and exclusion criteria of representative CHF trials with proportion of frail versus non-frail patients in our cohort who would not be eligible.

| CHF trials | - Frailty related inclusion/exclusion criteria | % patients excluded | | P (frail vs non-frail) |
| --- | --- | --- | --- | --- |
|  |  | Non-frail | Frail |  |
| DAPA-HF^[[1]](#endnote-1)^  Effect of Dapagliflozin on the Incidence of Worsening HF or CV Death in Patients With CHF | - - SBP <95 mmHg - - eGFR <30 mL/min/1.73 m^2^ | 5 | 19 | <0.001 |
| Paradigm HF^[[2]](#endnote-2)^  Efficacy and Safety of LCZ696 Compared to Enalapril on Morbidity and Mortality of Patients With CHF | - - SBP < 100 mmHg - - eGFR < 30 mL/min/1.73 m^2^ - - Serum K > 5.2 mmol/L - - ACEi/ ARB at a stable dose of at least enalapril 10mg/day for > 4 weeks | 65*  13** | 83*  25** | <0.001  0.001 |
| Paragon-HF^[[3]](#endnote-3)^  Efficacy and Safety of LCZ696 Compared to Valsartan, on Morbidity and Mortality in HF Patients With Preserved EF | - - Severe COPD - - Hb <10 g/dl - - BMI > 40 kg/m^2^ - - SBP ≥ 180 mmHg - - SBP < 110 mmHg | 43 | 58 | 0.001 |
| RALES^[[4]](#endnote-4)^  Effect of spironolactone on morbidity and mortality in patients with severe HF | - - Hepatic failure - - Active cancer - - Serum creatinine >221 µmol/L - - Serum K > 5.0 mmol/L | 31 | 37 | 0.18 |
| TOPCAT^[[5]](#endnote-5)^  Aldosterone Antagonist Therapy for Adults with HF and Preserved Systolic Function | - - Severe chronic pulmonary disease - - SBP > 160 mm Hg - - Serum K ≥ 5.5mmol/L - - eGFR <30 mL/min/1.73 m^2^ - - Severe hepatic disease | 46 | 65 | <0.001 |
| COMET^[[6]](#endnote-6)^  Comparison of carvedilol and metoprolol on clinical outcomes in patients with CHF | - - Asthma or COPD - - Insulin-dependent diabetes mellitus - - Severe hepatic disease - - HR < 60 bpm - SBP < 85 mm Hg | 38 | 54 | 0.001 |
| CHARM^[[7]](#endnote-7)^  Effects of candesartan in patients with CHF and reduced LV systolic function intolerant to ACEi | - Serum creatinine >265 µmol/L  - Serum K>5.5 mmol/L | 2 | 3 | 0.47 |

*considering all 4 criteria; **considering only the first 3 criteria

1. McMurray JJ, Solomon SD, Inzucchi SE, [Køber L](https://www.ncbi.nlm.nih.gov/pubmed/?term=K%C3%B8ber%20L%5BAuthor%5D&cauthor=true&cauthor_uid=31535829), [Kosiborod MN](https://www.ncbi.nlm.nih.gov/pubmed/?term=Kosiborod%20MN%5BAuthor%5D&cauthor=true&cauthor_uid=31535829), [Martinez FA](https://www.ncbi.nlm.nih.gov/pubmed/?term=Martinez%20FA%5BAuthor%5D&cauthor=true&cauthor_uid=31535829), [Ponikowski P](https://www.ncbi.nlm.nih.gov/pubmed/?term=Ponikowski%20P%5BAuthor%5D&cauthor=true&cauthor_uid=31535829), [Sabatine MS](https://www.ncbi.nlm.nih.gov/pubmed/?term=Sabatine%20MS%5BAuthor%5D&cauthor=true&cauthor_uid=31535829), [Anand IS](https://www.ncbi.nlm.nih.gov/pubmed/?term=Anand%20IS%5BAuthor%5D&cauthor=true&cauthor_uid=31535829), [Bělohlávek J](https://www.ncbi.nlm.nih.gov/pubmed/?term=B%C4%9Blohl%C3%A1vek%20J%5BAuthor%5D&cauthor=true&cauthor_uid=31535829), [Böhm M](https://www.ncbi.nlm.nih.gov/pubmed/?term=B%C3%B6hm%20M%5BAuthor%5D&cauthor=true&cauthor_uid=31535829), [Chiang CE](https://www.ncbi.nlm.nih.gov/pubmed/?term=Chiang%20CE%5BAuthor%5D&cauthor=true&cauthor_uid=31535829), [Chopra VK](https://www.ncbi.nlm.nih.gov/pubmed/?term=Chopra%20VK%5BAuthor%5D&cauthor=true&cauthor_uid=31535829), [de Boer RA](https://www.ncbi.nlm.nih.gov/pubmed/?term=de%20Boer%20RA%5BAuthor%5D&cauthor=true&cauthor_uid=31535829), [Desai AS](https://www.ncbi.nlm.nih.gov/pubmed/?term=Desai%20AS%5BAuthor%5D&cauthor=true&cauthor_uid=31535829), [Diez M](https://www.ncbi.nlm.nih.gov/pubmed/?term=Diez%20M%5BAuthor%5D&cauthor=true&cauthor_uid=31535829), [Drozdz J](https://www.ncbi.nlm.nih.gov/pubmed/?term=Drozdz%20J%5BAuthor%5D&cauthor=true&cauthor_uid=31535829), [Dukát A](https://www.ncbi.nlm.nih.gov/pubmed/?term=Duk%C3%A1t%20A%5BAuthor%5D&cauthor=true&cauthor_uid=31535829), [Ge J](https://www.ncbi.nlm.nih.gov/pubmed/?term=Ge%20J%5BAuthor%5D&cauthor=true&cauthor_uid=31535829), [Howlett JG](https://www.ncbi.nlm.nih.gov/pubmed/?term=Howlett%20JG%5BAuthor%5D&cauthor=true&cauthor_uid=31535829), [Katova T](https://www.ncbi.nlm.nih.gov/pubmed/?term=Katova%20T%5BAuthor%5D&cauthor=true&cauthor_uid=31535829), [Kitakaze M](https://www.ncbi.nlm.nih.gov/pubmed/?term=Kitakaze%20M%5BAuthor%5D&cauthor=true&cauthor_uid=31535829), [Ljungman CEA](https://www.ncbi.nlm.nih.gov/pubmed/?term=Ljungman%20CEA%5BAuthor%5D&cauthor=true&cauthor_uid=31535829), [Merkely B](https://www.ncbi.nlm.nih.gov/pubmed/?term=Merkely%20B%5BAuthor%5D&cauthor=true&cauthor_uid=31535829), [Nicolau JC](https://www.ncbi.nlm.nih.gov/pubmed/?term=Nicolau%20JC%5BAuthor%5D&cauthor=true&cauthor_uid=31535829), [O'Meara E](https://www.ncbi.nlm.nih.gov/pubmed/?term=O%27Meara%20E%5BAuthor%5D&cauthor=true&cauthor_uid=31535829), [Petrie MC](https://www.ncbi.nlm.nih.gov/pubmed/?term=Petrie%20MC%5BAuthor%5D&cauthor=true&cauthor_uid=31535829), [Vinh PN](https://www.ncbi.nlm.nih.gov/pubmed/?term=Vinh%20PN%5BAuthor%5D&cauthor=true&cauthor_uid=31535829), [Schou M](https://www.ncbi.nlm.nih.gov/pubmed/?term=Schou%20M%5BAuthor%5D&cauthor=true&cauthor_uid=31535829), [Tereshchenko S](https://www.ncbi.nlm.nih.gov/pubmed/?term=Tereshchenko%20S%5BAuthor%5D&cauthor=true&cauthor_uid=31535829), [Verma S](https://www.ncbi.nlm.nih.gov/pubmed/?term=Verma%20S%5BAuthor%5D&cauthor=true&cauthor_uid=31535829), [Held C](https://www.ncbi.nlm.nih.gov/pubmed/?term=Held%20C%5BAuthor%5D&cauthor=true&cauthor_uid=31535829), [DeMets DL](https://www.ncbi.nlm.nih.gov/pubmed/?term=DeMets%20DL%5BAuthor%5D&cauthor=true&cauthor_uid=31535829), [Docherty KF](https://www.ncbi.nlm.nih.gov/pubmed/?term=Docherty%20KF%5BAuthor%5D&cauthor=true&cauthor_uid=31535829), [Jhund PS](https://www.ncbi.nlm.nih.gov/pubmed/?term=Jhund%20PS%5BAuthor%5D&cauthor=true&cauthor_uid=31535829), [Bengtsson O](https://www.ncbi.nlm.nih.gov/pubmed/?term=Bengtsson%20O%5BAuthor%5D&cauthor=true&cauthor_uid=31535829), [Sjöstrand M](https://www.ncbi.nlm.nih.gov/pubmed/?term=Sj%C3%B6strand%20M%5BAuthor%5D&cauthor=true&cauthor_uid=31535829), [Langkilde AM](https://www.ncbi.nlm.nih.gov/pubmed/?term=Langkilde%20AM%5BAuthor%5D&cauthor=true&cauthor_uid=31535829); [DAPA-HF Trial Committees and Investigators](https://www.ncbi.nlm.nih.gov/pubmed/?term=DAPA-HF%20Trial%20Committees%20and%20Investigators%5BCorporate%20Author%5D). Dapagliflozin in Patients With Heart Failure and Reduced Ejection Fraction. [N Engl J Med 2019;381:1995-2008](https://www.nejm.org/doi/full/10.1056/NEJMoa1911303). [↑](#endnote-ref-1)
2. McMurray JJ, Packer M, Desai AS, Gong J, Lefkowitz MP, Rizkala AR, Rouleau JL, Shi VC, Solomon SD, Swedberg K, Zile MR; PARADIGM-HF Investigators and Committees.N Engl J Med. 2014;371:993-1004. [↑](#endnote-ref-2)
3. # [Solomon SD](https://www.ncbi.nlm.nih.gov/pubmed/?term=Solomon%20SD%5BAuthor%5D&cauthor=true&cauthor_uid=31475794), [McMurray JJV](https://www.ncbi.nlm.nih.gov/pubmed/?term=McMurray%20JJV%5BAuthor%5D&cauthor=true&cauthor_uid=31475794), [Anand IS](https://www.ncbi.nlm.nih.gov/pubmed/?term=Anand%20IS%5BAuthor%5D&cauthor=true&cauthor_uid=31475794), [Ge J](https://www.ncbi.nlm.nih.gov/pubmed/?term=Ge%20J%5BAuthor%5D&cauthor=true&cauthor_uid=31475794), [Lam CSP](https://www.ncbi.nlm.nih.gov/pubmed/?term=Lam%20CSP%5BAuthor%5D&cauthor=true&cauthor_uid=31475794), [Maggioni AP](https://www.ncbi.nlm.nih.gov/pubmed/?term=Maggioni%20AP%5BAuthor%5D&cauthor=true&cauthor_uid=31475794), [Martinez F](https://www.ncbi.nlm.nih.gov/pubmed/?term=Martinez%20F%5BAuthor%5D&cauthor=true&cauthor_uid=31475794), [Packer M](https://www.ncbi.nlm.nih.gov/pubmed/?term=Packer%20M%5BAuthor%5D&cauthor=true&cauthor_uid=31475794), [Pfeffer MA](https://www.ncbi.nlm.nih.gov/pubmed/?term=Pfeffer%20MA%5BAuthor%5D&cauthor=true&cauthor_uid=31475794), [Pieske B](https://www.ncbi.nlm.nih.gov/pubmed/?term=Pieske%20B%5BAuthor%5D&cauthor=true&cauthor_uid=31475794), [Redfield MM](https://www.ncbi.nlm.nih.gov/pubmed/?term=Redfield%20MM%5BAuthor%5D&cauthor=true&cauthor_uid=31475794), [Rouleau JL](https://www.ncbi.nlm.nih.gov/pubmed/?term=Rouleau%20JL%5BAuthor%5D&cauthor=true&cauthor_uid=31475794), [van Veldhuisen DJ](https://www.ncbi.nlm.nih.gov/pubmed/?term=van%20Veldhuisen%20DJ%5BAuthor%5D&cauthor=true&cauthor_uid=31475794), [Zannad F](https://www.ncbi.nlm.nih.gov/pubmed/?term=Zannad%20F%5BAuthor%5D&cauthor=true&cauthor_uid=31475794), [Zile MR](https://www.ncbi.nlm.nih.gov/pubmed/?term=Zile%20MR%5BAuthor%5D&cauthor=true&cauthor_uid=31475794), [Desai AS](https://www.ncbi.nlm.nih.gov/pubmed/?term=Desai%20AS%5BAuthor%5D&cauthor=true&cauthor_uid=31475794), [Claggett B](https://www.ncbi.nlm.nih.gov/pubmed/?term=Claggett%20B%5BAuthor%5D&cauthor=true&cauthor_uid=31475794), [Jhund PS](https://www.ncbi.nlm.nih.gov/pubmed/?term=Jhund%20PS%5BAuthor%5D&cauthor=true&cauthor_uid=31475794), [Boytsov SA](https://www.ncbi.nlm.nih.gov/pubmed/?term=Boytsov%20SA%5BAuthor%5D&cauthor=true&cauthor_uid=31475794), [Comin-Colet J](https://www.ncbi.nlm.nih.gov/pubmed/?term=Comin-Colet%20J%5BAuthor%5D&cauthor=true&cauthor_uid=31475794), [Cleland J](https://www.ncbi.nlm.nih.gov/pubmed/?term=Cleland%20J%5BAuthor%5D&cauthor=true&cauthor_uid=31475794), [Düngen HD](https://www.ncbi.nlm.nih.gov/pubmed/?term=D%C3%BCngen%20HD%5BAuthor%5D&cauthor=true&cauthor_uid=31475794), [Goncalvesova E](https://www.ncbi.nlm.nih.gov/pubmed/?term=Goncalvesova%20E%5BAuthor%5D&cauthor=true&cauthor_uid=31475794), [Katova T](https://www.ncbi.nlm.nih.gov/pubmed/?term=Katova%20T%5BAuthor%5D&cauthor=true&cauthor_uid=31475794), [Kerr Saraiva JF](https://www.ncbi.nlm.nih.gov/pubmed/?term=Kerr%20Saraiva%20JF%5BAuthor%5D&cauthor=true&cauthor_uid=31475794), [Lelonek M](https://www.ncbi.nlm.nih.gov/pubmed/?term=Lelonek%20M%5BAuthor%5D&cauthor=true&cauthor_uid=31475794), [Merkely B](https://www.ncbi.nlm.nih.gov/pubmed/?term=Merkely%20B%5BAuthor%5D&cauthor=true&cauthor_uid=31475794), [Senni M](https://www.ncbi.nlm.nih.gov/pubmed/?term=Senni%20M%5BAuthor%5D&cauthor=true&cauthor_uid=31475794), [Shah SJ](https://www.ncbi.nlm.nih.gov/pubmed/?term=Shah%20SJ%5BAuthor%5D&cauthor=true&cauthor_uid=31475794), [Zhou J](https://www.ncbi.nlm.nih.gov/pubmed/?term=Zhou%20J%5BAuthor%5D&cauthor=true&cauthor_uid=31475794), [Rizkala AR](https://www.ncbi.nlm.nih.gov/pubmed/?term=Rizkala%20AR%5BAuthor%5D&cauthor=true&cauthor_uid=31475794), [Gong J](https://www.ncbi.nlm.nih.gov/pubmed/?term=Gong%20J%5BAuthor%5D&cauthor=true&cauthor_uid=31475794), [Shi VC](https://www.ncbi.nlm.nih.gov/pubmed/?term=Shi%20VC%5BAuthor%5D&cauthor=true&cauthor_uid=31475794), [Lefkowitz MP](https://www.ncbi.nlm.nih.gov/pubmed/?term=Lefkowitz%20MP%5BAuthor%5D&cauthor=true&cauthor_uid=31475794); [PARAGON-HF Investigators and Committees](https://www.ncbi.nlm.nih.gov/pubmed/?term=PARAGON-HF%20Investigators%20and%20Committees%5BCorporate%20Author%5D). Angiotensin-Neprilysin Inhibition in Heart Failure with Preserved Ejection Fraction. [N Engl J Med.](https://www.ncbi.nlm.nih.gov/pubmed/31475794) 2019;381:1609-1620.

   [↑](#endnote-ref-3)
4. # [Pitt B](https://www.ncbi.nlm.nih.gov/pubmed/?term=Pitt%20B%5BAuthor%5D&cauthor=true&cauthor_uid=10471456), [Zannad F](https://www.ncbi.nlm.nih.gov/pubmed/?term=Zannad%20F%5BAuthor%5D&cauthor=true&cauthor_uid=10471456), [Remme WJ](https://www.ncbi.nlm.nih.gov/pubmed/?term=Remme%20WJ%5BAuthor%5D&cauthor=true&cauthor_uid=10471456), [Cody R](https://www.ncbi.nlm.nih.gov/pubmed/?term=Cody%20R%5BAuthor%5D&cauthor=true&cauthor_uid=10471456), [Castaigne A](https://www.ncbi.nlm.nih.gov/pubmed/?term=Castaigne%20A%5BAuthor%5D&cauthor=true&cauthor_uid=10471456), [Perez A](https://www.ncbi.nlm.nih.gov/pubmed/?term=Perez%20A%5BAuthor%5D&cauthor=true&cauthor_uid=10471456), [Palensky J](https://www.ncbi.nlm.nih.gov/pubmed/?term=Palensky%20J%5BAuthor%5D&cauthor=true&cauthor_uid=10471456), [Wittes J](https://www.ncbi.nlm.nih.gov/pubmed/?term=Wittes%20J%5BAuthor%5D&cauthor=true&cauthor_uid=10471456). The effect of spironolactone on morbidity and mortality in patients with severe heart failure. Randomized Aldactone Evaluation Study Investigators. [N Engl J Med.](https://www.ncbi.nlm.nih.gov/pubmed/10471456) 1999;341:709-17.

   [↑](#endnote-ref-4)
5. # [Pitt B](https://www.ncbi.nlm.nih.gov/pubmed/?term=Pitt%20B%5BAuthor%5D&cauthor=true&cauthor_uid=24716680), [Pfeffer MA](https://www.ncbi.nlm.nih.gov/pubmed/?term=Pfeffer%20MA%5BAuthor%5D&cauthor=true&cauthor_uid=24716680), [Assmann SF](https://www.ncbi.nlm.nih.gov/pubmed/?term=Assmann%20SF%5BAuthor%5D&cauthor=true&cauthor_uid=24716680), [Boineau R](https://www.ncbi.nlm.nih.gov/pubmed/?term=Boineau%20R%5BAuthor%5D&cauthor=true&cauthor_uid=24716680), [Anand IS](https://www.ncbi.nlm.nih.gov/pubmed/?term=Anand%20IS%5BAuthor%5D&cauthor=true&cauthor_uid=24716680), [Claggett B](https://www.ncbi.nlm.nih.gov/pubmed/?term=Claggett%20B%5BAuthor%5D&cauthor=true&cauthor_uid=24716680), [Clausell N](https://www.ncbi.nlm.nih.gov/pubmed/?term=Clausell%20N%5BAuthor%5D&cauthor=true&cauthor_uid=24716680), [Desai AS](https://www.ncbi.nlm.nih.gov/pubmed/?term=Desai%20AS%5BAuthor%5D&cauthor=true&cauthor_uid=24716680), [Diaz R](https://www.ncbi.nlm.nih.gov/pubmed/?term=Diaz%20R%5BAuthor%5D&cauthor=true&cauthor_uid=24716680), [Fleg JL](https://www.ncbi.nlm.nih.gov/pubmed/?term=Fleg%20JL%5BAuthor%5D&cauthor=true&cauthor_uid=24716680), [Gordeev I](https://www.ncbi.nlm.nih.gov/pubmed/?term=Gordeev%20I%5BAuthor%5D&cauthor=true&cauthor_uid=24716680), [Harty B](https://www.ncbi.nlm.nih.gov/pubmed/?term=Harty%20B%5BAuthor%5D&cauthor=true&cauthor_uid=24716680), [Heitner JF](https://www.ncbi.nlm.nih.gov/pubmed/?term=Heitner%20JF%5BAuthor%5D&cauthor=true&cauthor_uid=24716680), [Kenwood CT](https://www.ncbi.nlm.nih.gov/pubmed/?term=Kenwood%20CT%5BAuthor%5D&cauthor=true&cauthor_uid=24716680), [Lewis EF](https://www.ncbi.nlm.nih.gov/pubmed/?term=Lewis%20EF%5BAuthor%5D&cauthor=true&cauthor_uid=24716680), [O'Meara E](https://www.ncbi.nlm.nih.gov/pubmed/?term=O%27Meara%20E%5BAuthor%5D&cauthor=true&cauthor_uid=24716680), [Probstfield JL](https://www.ncbi.nlm.nih.gov/pubmed/?term=Probstfield%20JL%5BAuthor%5D&cauthor=true&cauthor_uid=24716680), [Shaburishvili T](https://www.ncbi.nlm.nih.gov/pubmed/?term=Shaburishvili%20T%5BAuthor%5D&cauthor=true&cauthor_uid=24716680), [Shah SJ](https://www.ncbi.nlm.nih.gov/pubmed/?term=Shah%20SJ%5BAuthor%5D&cauthor=true&cauthor_uid=24716680), [Solomon SD](https://www.ncbi.nlm.nih.gov/pubmed/?term=Solomon%20SD%5BAuthor%5D&cauthor=true&cauthor_uid=24716680), [Sweitzer NK](https://www.ncbi.nlm.nih.gov/pubmed/?term=Sweitzer%20NK%5BAuthor%5D&cauthor=true&cauthor_uid=24716680), [Yang S](https://www.ncbi.nlm.nih.gov/pubmed/?term=Yang%20S%5BAuthor%5D&cauthor=true&cauthor_uid=24716680), [McKinlay SM](https://www.ncbi.nlm.nih.gov/pubmed/?term=McKinlay%20SM%5BAuthor%5D&cauthor=true&cauthor_uid=24716680); [TOPCAT Investigators](https://www.ncbi.nlm.nih.gov/pubmed/?term=TOPCAT%20Investigators%5BCorporate%20Author%5D). Spironolactone for heart failure with preserved ejection fraction.[N Engl J Med.](https://www.ncbi.nlm.nih.gov/pubmed/24716680) 2014;370:1383-92.

   [↑](#endnote-ref-5)
6. # [Poole-Wilson PA](https://www.ncbi.nlm.nih.gov/pubmed/?term=Poole-Wilson%20PA%5BAuthor%5D&cauthor=true&cauthor_uid=12853193), [Swedberg K](https://www.ncbi.nlm.nih.gov/pubmed/?term=Swedberg%20K%5BAuthor%5D&cauthor=true&cauthor_uid=12853193), [Cleland JG](https://www.ncbi.nlm.nih.gov/pubmed/?term=Cleland%20JG%5BAuthor%5D&cauthor=true&cauthor_uid=12853193), [Di Lenarda A](https://www.ncbi.nlm.nih.gov/pubmed/?term=Di%20Lenarda%20A%5BAuthor%5D&cauthor=true&cauthor_uid=12853193), [Hanrath P](https://www.ncbi.nlm.nih.gov/pubmed/?term=Hanrath%20P%5BAuthor%5D&cauthor=true&cauthor_uid=12853193), [Komajda M](https://www.ncbi.nlm.nih.gov/pubmed/?term=Komajda%20M%5BAuthor%5D&cauthor=true&cauthor_uid=12853193), [Lubsen J](https://www.ncbi.nlm.nih.gov/pubmed/?term=Lubsen%20J%5BAuthor%5D&cauthor=true&cauthor_uid=12853193), [Lutiger B](https://www.ncbi.nlm.nih.gov/pubmed/?term=Lutiger%20B%5BAuthor%5D&cauthor=true&cauthor_uid=12853193), [Metra M](https://www.ncbi.nlm.nih.gov/pubmed/?term=Metra%20M%5BAuthor%5D&cauthor=true&cauthor_uid=12853193), [Remme WJ](https://www.ncbi.nlm.nih.gov/pubmed/?term=Remme%20WJ%5BAuthor%5D&cauthor=true&cauthor_uid=12853193), [Torp-Pedersen C](https://www.ncbi.nlm.nih.gov/pubmed/?term=Torp-Pedersen%20C%5BAuthor%5D&cauthor=true&cauthor_uid=12853193), [Scherhag A](https://www.ncbi.nlm.nih.gov/pubmed/?term=Scherhag%20A%5BAuthor%5D&cauthor=true&cauthor_uid=12853193), [Skene A](https://www.ncbi.nlm.nih.gov/pubmed/?term=Skene%20A%5BAuthor%5D&cauthor=true&cauthor_uid=12853193); [Carvedilol Or Metoprolol European Trial Investigators](https://www.ncbi.nlm.nih.gov/pubmed/?term=Carvedilol%20Or%20Metoprolol%20European%20Trial%20Investigators%5BCorporate%20Author%5D). Comparison of carvedilol and metoprolol on clinical outcomes in patients with chronic heart failure in the Carvedilol Or Metoprolol European Trial (COMET): randomised controlled trial. [Lancet.](https://www.ncbi.nlm.nih.gov/pubmed/12853193) 2003;362:7-13.

   [↑](#endnote-ref-6)
7. # [Pfeffer MA](https://www.ncbi.nlm.nih.gov/pubmed/?term=Pfeffer%20MA%5BAuthor%5D&cauthor=true&cauthor_uid=13678868), [Swedberg K](https://www.ncbi.nlm.nih.gov/pubmed/?term=Swedberg%20K%5BAuthor%5D&cauthor=true&cauthor_uid=13678868), [Granger CB](https://www.ncbi.nlm.nih.gov/pubmed/?term=Granger%20CB%5BAuthor%5D&cauthor=true&cauthor_uid=13678868), [Held P](https://www.ncbi.nlm.nih.gov/pubmed/?term=Held%20P%5BAuthor%5D&cauthor=true&cauthor_uid=13678868), [McMurray JJ](https://www.ncbi.nlm.nih.gov/pubmed/?term=McMurray%20JJ%5BAuthor%5D&cauthor=true&cauthor_uid=13678868), [Michelson EL](https://www.ncbi.nlm.nih.gov/pubmed/?term=Michelson%20EL%5BAuthor%5D&cauthor=true&cauthor_uid=13678868), [Olofsson B](https://www.ncbi.nlm.nih.gov/pubmed/?term=Olofsson%20B%5BAuthor%5D&cauthor=true&cauthor_uid=13678868), [Ostergren J](https://www.ncbi.nlm.nih.gov/pubmed/?term=Ostergren%20J%5BAuthor%5D&cauthor=true&cauthor_uid=13678868), [Yusuf S](https://www.ncbi.nlm.nih.gov/pubmed/?term=Yusuf%20S%5BAuthor%5D&cauthor=true&cauthor_uid=13678868), [Pocock S](https://www.ncbi.nlm.nih.gov/pubmed/?term=Pocock%20S%5BAuthor%5D&cauthor=true&cauthor_uid=13678868); [CHARM Investigators and Committees](https://www.ncbi.nlm.nih.gov/pubmed/?term=CHARM%20Investigators%20and%20Committees%5BCorporate%20Author%5D). Effects of candesartan on mortality and morbidity in patients with chronic heart failure: the CHARM-Overall programme. [Lancet.](https://www.ncbi.nlm.nih.gov/pubmed/13678868) 2003;362:759-66.

   [↑](#endnote-ref-7)
